# An Impermeant Ganetespib Analog Inhibits Extracellular Hsp90-Mediated Cancer Cell Migration that Involves Lysyl Oxidase 2-like Protein

**DOI:** 10.3390/cancers6021031

**Published:** 2014-04-30

**Authors:** Jessica McCready, Daniel S. Wong, Joseph A. Burlison, Weiwen Ying, Daniel G. Jay

**Affiliations:** 1 Department of Natural Sciences, Assumption College, Worcester, MA 01609, USA; E-Mail: j.mccready@assumption.edu; 2 Department of Developmental Molecular and Chemical Biology, Tufts University School of Medicine, Boston, MA 02111, USA; E-Mail: daniel_s.wong@tufts.edu; 3 Cell and Molecular Physiology Program, Sackler School of Graduate Biomedical Sciences, Tufts University, Boston, MA 02111, USA; 4 Synta Pharmaceuticals, Lexington, MA 02421, USA; E-Mails: joeburlison@gmail.com (J.A.B.); wying@syntapharma.com (W.Y.)

**Keywords:** extracellular Hsp90, ganetespib, lysyl oxidase 2-like protein, cancer cell migration

## Abstract

Extracellular Hsp90 (eHsp90) activates a number of client proteins outside of cancer cells required for migration and invasion. Therefore, eHsp90 may serve as a novel target for anti-metastatic drugs as its inhibition using impermeant Hsp90 inhibitors would not affect the numerous vital intracellular Hsp90 functions in normal cells. While some eHsp90 clients are known, it is important to establish other proteins that act outside the cell to validate eHsp90 as a drug target to limit cancer spread. Using mass spectrometry we identified two precursor proteins Galectin 3 binding protein (G3BP) and Lysyl oxidase 2-like protein (LOXL2) that associate with eHsp90 in MDA-MB231 breast cancer cell conditioned media and confirmed that LOXL2 binds to eHsp90 in immunoprecipitates. We introduce a novel impermeant Hsp90 inhibitor STA-12-7191 derived from ganetespib and show that it is markedly less toxic to cells and can inhibit cancer cell migration in a dose dependent manner. We used STA-12-7191 to test if LOXL2 and G3BP are potential eHsp90 clients. We showed that while LOXL2 can increase wound healing and compensate for STA-12-7191-mediated inhibition of wound closure, addition of G3BP had no affect on this assay. These findings support of role for LOXL2 in eHsp90 stimulated cancer cell migration and provide preliminary evidence for the use of STA-12-7191 to inhibit eHsp90 to limit cancer invasion.

## 1. Introduction

The vast majority of cancer related deaths result not from the primary tumor, but from metastasis. There are currently no anti-metastasis drugs and developing such drugs would address an important unmet need in cancer treatment. Metastasis is a complex multi-step process initiated by invasion of tumor cells into the extra-tumor environment, extravasation into the circulatory or lymphatic system, survival of these cells while circulating and homing and invasion into a secondary site and finally survival and proliferation in the new environment [1]. While interfering with any of these steps would be useful, we reasoned that invasion as a first step in metastasis would have promise in containing tumors.

To identify novel proteins that act in invasiveness, we applied a functional proteomic screen using Fluorophore-Assisted Light Inactivation targeted with antibody libraries selected against proteins expressed on the surface of HT-1080 fibrosarcoma cells [2] This screen revealed three proteins newly implicated in invasion [3,4] and the most intriguing is extracellular Hsp90 (eHsp90). Hsp90 is a highly abundant intracellular protein that serves as a molecular chaperone for hundreds of client protein inside of cells [5,6] but demonstration of its role outside of cells had been limited [7]. Our study demonstrated that the alpha (but not beta) isoform of Hsp90 is found on the surface of HT1080 fibrosarcoma cells and that its inhibition reduces cell invasiveness and migration [2]. Moreover, eHsp90 activated the matrix metalloproteinase 2 (MMP2) by processing pro-MMP2 and the reduced invasion by specific inhibition of eHsp90 could be rescued by addition of active MMP2 to the cells.

Since our initial discovery of eHsp90 in cancer invasion [2], there have been numerous publications implicating eHsp90 in cancer and other diseases and have identified new extracellular clients and cellular processes impacted by eHsp90 [8] especially in cancers such as breast [9,10] colorectal [11] GBM [12], and prostate [13]. In addition to our work on MMP2, others have shown the following to be eHsp90 clients: MMP9 [14]; human EGF receptor 2 (HER2 [9]; and LDL Receptor-like protein (LRP-1 [13,15]; which are all involved in cancer invasiveness and other disease-relevant processes [8]. For example, to identify binders of eHsp90 we used mass spectrometry analysis of Hsp90 alpha immunoprecipitation from MB-231 breast cancer cell conditioned media to identify a pro-form of tissue plasminogen activator (TPA) and then showed that active TPA could rescue eHsp90 inhibition-mediated loss of cell migration [10]. Together, these studies have shown important roles for eHsp90 in cell migration and ECM remodeling [10,14], and epithelial to mesenchymal transition [13]. Additionally inhibition of eHsp90 in mouse models for metastasis have shown benefit in survival and reduced metastasis suggesting the clinical potential of inhibiting the extracellular roles of Hsp90 without affecting its intracellular functions.

While these studies have expanded the repertoire of eHsp90 clients, our understanding of its novel cancer relevant processes remains incomplete. Identifying and validating new extracellular clients for eHsp90 and determining their roles in cancer progression and metastasis will inform optimal design of eHsp90 drugs for reducing metastasis and establishing a serum biomarker for drug efficacy. In this paper, we report additional mass spectrometry analysis of Hsp90 binders in conditioned media and we test if eHsp90’s role in enhancing cell migration (a critical component of invasion) has any contribution from these binders using a novel impermeant Hsp90 inhibitor derived from ganetespib, a promising drug currently in clinical trials [16,17].

## 2. Results and Discussion

### 2.1. Mass Spectrometry Analysis of Hsp90 Binders from Conditioned Media

In addition to TPA [10], several other pro-proteins were identified as eHsp90 binders from MDA-MB231 conditioned media. Mass spectrometry analysis indicates that the protein with the highest number of peptides following trypsin digestion (Table 1) was Galectin-3 binding protein (G3BP), followed by the precursor form of Lysyl Oxidase-like 2 protein (LOXL2) (Table 1). G3BP and LOXL2 are both interesting candidates for eHsp90 activation because of their roles in cancer. G3BP (also called Mac2 Binding Protein) was identified as a secreted glycoprotein in macrophages [18] and found in human breast carcinoma and in breast milk [19]. It has been extensively implicated in many types of cancer. Specifically, it is a biomarker in breast cancer sera and proximal fluid [20,21] as well as in colon cancer [22]. Its presence in non-small cell lung carcinoma patients predicts poor survival and metastasis [23]. LOXL2 regulates cell polarity in breast carcinoma and is required for metastasis [24]. LOXL2 has been implicated in breast carcinoma [25] and its overexpression is associated with poor clinical outcome [26] and metastasis [27] for breast adenocarcinomas.

**Table 1:** Mass spectrometry reveals novel binders of eHsp90.

Peptide	Score	Peptide (*Hits)
Heat Shock Protein 90 kDa alpha (cytosolic), class A member 1 isoform 2	288.34	112 (108 4 0 0 0)
**Galectin-3 Binding Protein**	**210.3**	**135 (135 0 0 0 0)**
**Lysyl Oxidase-like 2 precursor**	**70.26**	**10 (10 0 0 0)**
Heat Shock Protein-1 beta	68.23	15 (10 5 0 0 0)
Lysyl Oxidase-like 4 precursor	48.19	6 (4 2 0 0 0)
Plasminogen Activator, tissue type isoform 1 preprotein	40.26	5 (5 0 0 0 0)
Granulin precursor	40.23	5 (5 0 0 0 0)
MAM domain containing 2	40.22	5 (5 0 0 0 0)
Galactosylceramidase isoform a precurso	30.26	3 (3 0 0 0 0)
Albumin precursor	30.23	4 (4 0 0 0 0)

* indicates the number of peptides in each sample following trypsin digestion.

**Figure 1:**
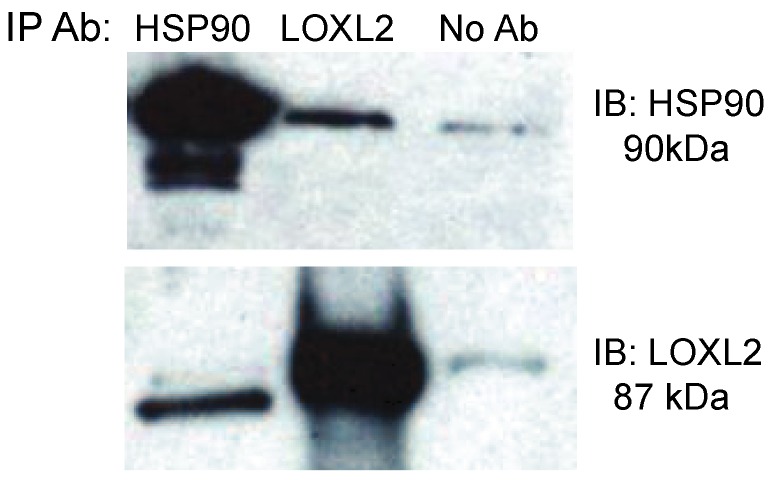
eHsp90 binds to LOXL2 in conditioned media of breast cancer cells. Immunoprecipitation of hsp90 or LOXL2 from the conditioned media of MDA-MB231 cells. Immunoprecipitation antibody (IP Ab) is indicated along the top row while the antibody used to probe the Western blot (IB) is indicated along the right side of the figure.

We confirmed that LOXL2 binds eHsp90 by immunoblot analysis of immunoprecipitates both using anti-Hsp90 alpha followed by immunoblot with anti-LOXL2 as well as using anti-LOXL2 for coimmunoprecipitation followed by immunoblotting using anti-Hsp90 alpha (Figure 1). In contrast, we were unable to detect G3BP by this approach (data not shown). These findings suggest that LOXL2 (precursor) associates with Hsp90 alpha in conditioned media while G3BP precursor may be part of a complex with aggregates of extracellular matrix that nonspecifically precipitate when immunoprecipitating eHsp90. This may be due to G3BP’s ability to bind to the extracellular matrix and integrins or its binding to Galectin-3 [28,29].

Interestingly, Hsp90 beta was identified as a binder of Hsp90 alpha in MDA-MB231 conditioned media. Our initial studies using HT-1080 fibrosarcoma cells showed that only the alpha isoform was located on the HT1080 cell surface by immunohistochemistry [2]. This was an argument that eHsp90 was not due to leakage of intracellular content but was specific. Since then however, several papers have shown alpha, beta or both isoforms of Hsp90 in the extsracellular environment [11,14,30]. For example, in MDA-MB453 cells have both isoforms in their conditioned media [14]*.* Our findings suggest that for MDA-MB231 cells that both isoforms are also present and we speculate that the isoform specificity may be cell-type dependent.

### 2.2. Inhibiting eHsp90 Using STA-12-7191: An Impermeant Derivative of Ganetespib

The singular targeting of eHsp90 is likely to inhibit many of its clients whose activities promote invasion leading to a more marked effect on metastasis compared to inhibition of any one of these proteins alone. Studies using mouse models of metastasis have indicated that inhibition of eHsp90 using DMAG N-oxide or an inhibiting monoclonal antibody 4c5 reduced metastases [14,31]. Despite this promise, there are issues that limit the use of these inhibitors for drug development. DMAG N-oxide generates a metabolic product that can cause retinal damage while 4c5 is a large protein that may have concerns with tumor penetrance [32]. Recently, a tethered (and thus impermeant) Hsp90 inhibitor (HS-27) has been reported [33] but it has not yet been tested clinically. Data presented herein introduce a novel impermeant small molecule Hsp90 inhibitor STA-12-7191 derived from the drug ganetespib, which has been tested in clinical trials [17]. Permeability assays show that STA-12-7191 has a ranked permeability of 0.02 × 10^−8^ cm/s (Table 2). In contrast, ganetespib has a permeability ranking of 3.6 × 10^−6^ cm/s. Permeable compounds must have a ranking in the order of 10^−6^ cm/s. Thus, STA-12-7191 is not only cell impermeant but also markedly less permeable than ganetespib.

**Table 2:** Permeability data for Hsp90 inhibitors indicates that STA-12-7191 is membrane impermeant.

Compound	Permeability	
	(cm/s)	(10E−06 cm/s)
STA-12-7191	2.10E−08	0.021
Ganetespib	3.6E−06	3.6
Caffeine (+)	1.17E−05	11.7
Furosemide (−)	1.47E−08	0.0147

### 2.3. STA-12-7191 Is Markedly Less Toxic than Ganetespib

**Figure 2:**
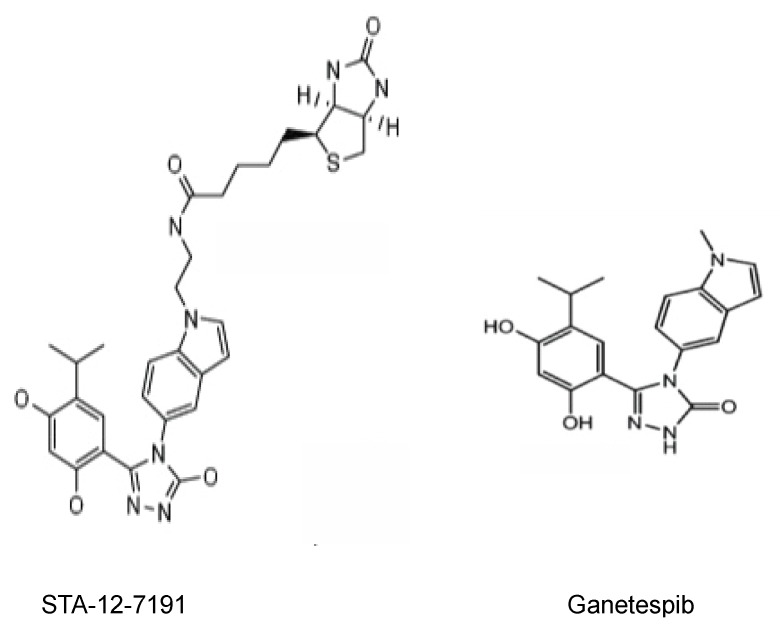
Structure of HSP90 inhibitors. The chemical structures for ganetespib and for STA-12-7191, a biotinylated derivative of ganetespib.

STA-12-7191 is a biotinylated analog of ganetespib (Figure 2). Ganetespib binds to the ATP binding site of Hsp90 alpha with a K_d_ of 110 nM [34] and we measured this binding for STA-7191 using an assay for labeled geldanamycin competition and measured an IC_50_ of 62 nM showing that it still binds tightly to the ATP binding site comparable to ganetespib itself. We postulate that STA-12-7191 does not readily penetrate the cell membrane due to the polar biotin moiety. This is shown by the 100-fold difference in the IC_50_ for its inhibition HER2 degradation, an intracellular function of Hsp90. STA-12-7191 had an EC_50_ for HER2 degradation of 2.7 µM in BT-474 breast cancer cells compared to an EC_50_ of 29 nM for ganetespib (Table 3).

**Table 3:** EC_50_ for Geldanomycin competition and HER2 degradation for ganetespib and STA-12-7191.

	Geldanamycin binding EC50	Her2 degradation EC50
Ganetespib	110 nM	29 nM
STA-12-7191	62 nM	2557 nM

**Figure 3:**
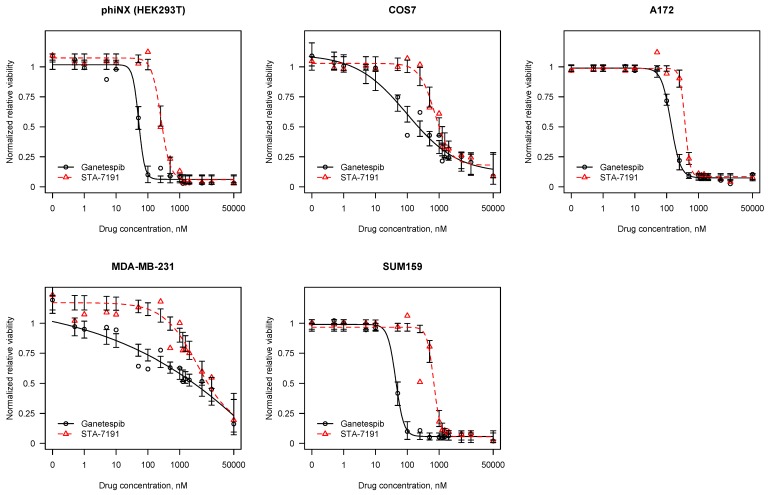
STA-12-7191 is markedly less toxic than ganetespib. CellTiterGlo was used to assess viability of HEK293T, MDA-MB231 and A172 cells after treatment with varying concentrations of either ganetespib (black) or STA-12-7191 for 5 days. Data are means ± SEM and representative of two independent experiments. LD50 values were determined using the drc package in R statistical software.

We first tested the effects of STA-12-7191 on cell viability on both cancer and non-cancer cells (Figure 3). Interestingly, the LD_50_ values varied markedly depending on the cell line tested. HEK293T and A172 cells were more sensitive to both drugs than were MDA-MB231 cells. STA-12-7191 has a 6-fold higher LD_50_ compared with ganetespib in HEK293T cells (54 nM *vs.* 306 nM), as we expect due to its reduced ability to cross the membrane. This is consistent with the difference between EC_50_ for geldanamycin binding and HER2 degradation for these two drugs shown in Table 3. This supports the notion that inhibiting eHsp90 is not toxic to normal cells and as a drug candidate might be tolerated at higher concentrations than ganetespib. We also observed a difference for LD_50_ between ganetespib and STA-12-7191 for A172 Glioblastoma cells though not as large as seen for HEK293T cells (157 nM *vs.* 387 nM). The LD_50_ for STA-12-7191 for HEK293T and A172 cells are similar but the LD_50_ for ganetespib is three fold higher perhaps due to drug resistance mechanisms. Interestingly for MDA-MB231 breast cancer cells while we again noticed a similar difference in LD_50_ for the two drugs this cell type is markedly more resistant to both drugs with LD_50_ values in µM range (2.54 µM *vs.* 9.34 µM). This is likely due to the high drug resistance inherent in these cells due to high expression of the multi-drug resistance pump, PGP-1 [35].

As STA12-7191 is thought to be impermeant, the question is why it is toxic at all? Barrott *et al*. [6], have recently shown that tethered Hsp90 inhibitors can enter cells through an endocytic process mediated by eHsp90. We speculate that STA-12-7191 also enters the cell via this process (or perhaps passive or active transport) albeit slowly relative to ganetespib leading to cell toxicity. This is consistent with the fact that STA-12-7191 inhibits HER2 degradation with a much higher IC_50_ than ganetespib.

### 2.4. Testing STA-12-7191 to Inhibit eHsp90-Mediated Cell Migration

**Figure 4:**
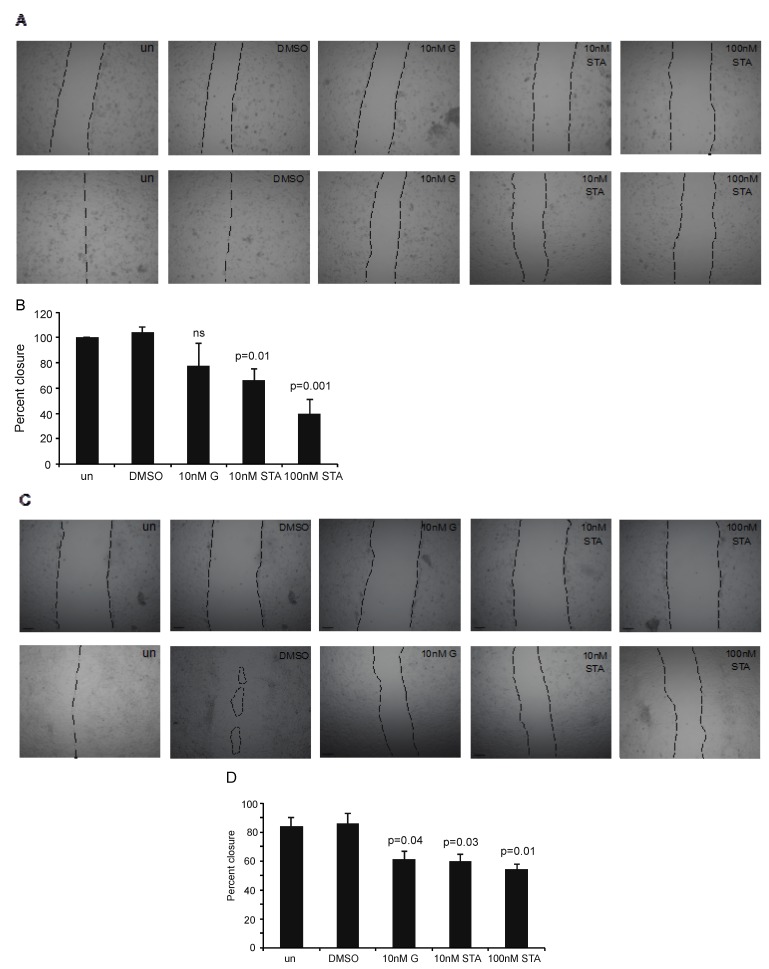
Inhibition of extracellular hsp90 reduces migration of breast cancer and brain tumor cell lines. (**A**) Representative images of MDA-MB231 breast cancer cells at 0 h (top panel) or 16 h (bottom panel) post wounding. Cells were treated with DMSO, Ganetespib (G), or STA-1791 (STA). Dotted lines outline the width of the wound unoccupied by cells; (**B**) Quantification of wound healing assay using MDA-MB231 cells. Data are represented as the percent closure relative to untreated cells (un). *p* value indicates significance compared to DMSO treated cells (two tailed t test). Data are means ± SEM; *n* = 4 experiments; (**C**) Representative images of A172 glioma cells at 0 h (top panel) or 16 h(bottom panel) post wounding. Cells were treated with DMSO, Ganetespib (G), or STA-1791 (STA). Dotted lines outline the width of the wound sunoccupied by cells; (**D**) Quantification of wound healing assay using A172 cells. Data are represented as the percent closure at 16 h relative to 0 h for each treatment. *p* value indicates significance compared to DMSO treated cells (two tailed t test). Data are means ± SEM; *n* = 3 experiments.

We tested and compared ganetespib and STA-12-7191 to control for their efficacy in reducing cancer cell motility as assayed using wound healing assays. We test this using two cancer cell lines, MDA-MB231 breast cancer cells and A172 GBM cells. These cell lines were selected as highly invasive cancer cell lines based on cancers that have been shown to highly express eHsp90 [10] and that treatment with eHsp90 specific inhibitors (DMAG-oxide and 4c5) resulted in modest benefit in reducing metastasis. Lastly, we had previously used these cell lines to show eHsp90’s role in cell migration in culture [10]. We chose concentrations of 10 nM and 100 nM concentrations that bracket the IC_50_ for STA-12-7191 inhibition of geldanamycin binding (62 nM) but still markedly below the IC_50_ for HER2 degradation (620 nM) to minimize potential effects on intracellular Hsp90 functions by STA-12-7191. For MDA-MB231 cells, treatment with 10 nM ganetespib and STA-12-7191 blocked wound healing by 20% (Figure 4A,B). We also treated cells with 100 nM ganetespib which is below its LD_50_ for all cells tested, however the cells appeared to round up and became non-motile (data not shown). Therefore, we could not conclude if cell migration was inhibited due to this behavior or an effect on their viability. In contrast treatment of MDA-MB231 cells with 10 nM STA-12-7191 resulted in 30% inhibition of wound closure (*p* = 0.01 and treatment with 100 nM STA-12-7191 did not affect cell morphology and resulted in close to 60% inhibition of cell migration. This result was also seen using A172 GBM cells though no increased inhibition was seen at 100 nM STA-12-7191 (Figure 4C,D).

These findings show that STA-7191 can inhibit cancer cell migration and can be used at higher concentrations that cannot be used for ganetespib. The fact that ganetespib was no better at inhibiting cell migration at 10 nM than STA-12-7191 suggests that the major role of Hsp90 on cell migration in this assay is based on its extracellular activity. The viability and wound healing data make a key point that at concentrations of STA-12-7191 that cells can tolerate it has a greater effect on migration while we can go up in STA-12-7191 beyond the tolerable dose for ganetespib and cause an even greater loss of cell migration. Interestingly, even 10 nM exceeds the K_d_ for ganetespib binding to the Hsp90-ATPase site suggesting perhaps a different mechanism beyond the ATPase binding pocket inhibition. It is also curious why an inhibitor that acts at this site should inhibit migration as we showed that ATP hydrolysis was not required for MMP2 activation by eHsp90 [36]. In fact, a non-hydrolysable form of ATP (gamma S) actually caused increased MMP2 activation. We speculate that while ATPase activity is not needed for eHsp90 activation of its clients, perhaps ATP binding facilitates conformation that enhances its action on its extracellular partners.

### 2.5. LOXL2 Can Rescue of STA-12-7191 Inhibition of Cell Migration

**Figure 5:**
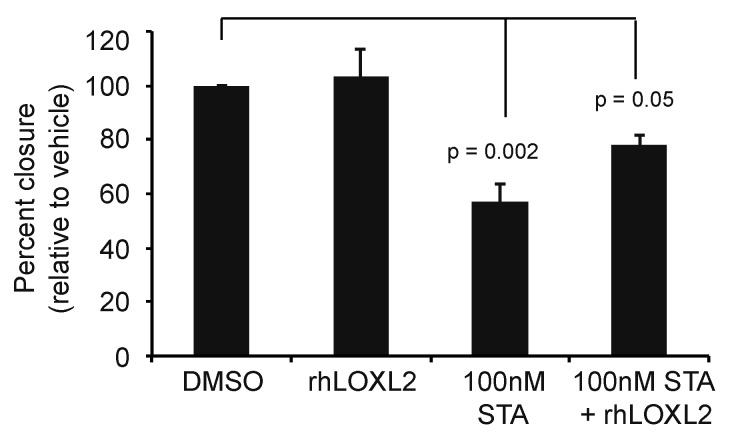
Role of eHsp90 on breast cancer cell motility is dependent upon. LOXL2. Quantification of wound healing assay using MDA-MB231 cells. Data are represented as the percent closure relative to vehicle treated cells (DMSO). Cells were treated with DMSO, 50 ng recombinant human LOXL2 protein (LOXL2), 100 nM STA-12-7191 (STA), or the combination of both recombinant human LOXL2 protein with 100nM STA-12-7191. *p* values calculated using two tailed t test. Data are means ± SEM; *n* = 3 experiments.

We addressed whether LOXL2, the novel eHsp90 binder that we found, has a role in eHsp90-mediated cell migration using the wound healing assay. The decrease of wound closure caused by 100 nM STA-12-7191 can be rescued by adding LOXL2 protein though not entirely (Figure 5). This is not surprising as there are many eHsp90 clients that act in cell migration including MMP2, MMP9, and TPA among others. We similarly tested G3BP for its role in wound healing and saw no significant effect of adding purified G3BP regardless of STA-12-7191 (data not shown).

## 3. Experimental

### 3.1. Cell Culture

HEK293T, A172, COS7, and MDA-MB231 cells were obtained from ATCC and maintained in DMEM supplemented with 10% FBS, 1% NEAA and 1% P/S. SUM159 cells were a kind gift from Dr. Charlotte Kuperwasser (Tufts Univsersity School of Medicine, Boston, MA, USA) and were maintained in F-12 media supplemented with 5% FBS, 5 μg/mL insulin, 0.5 μg/mL hydrocortisone and 1% P/S. All cells were grown in a 37 °C incubator with 5% CO_2_.

### 3.2. Immunoprecipitation/Mass Spectrometry

Immunoprecipitation and mass spectrometry were performed as described in [10]. 4 × 10^6^ MDA-MB231 breast cancer cells were plated in a 150 mm tissue culture dish and allowed to settle for 24 h. Cells were then re-fed with serum free media and incubated for 48 h at 37 °C. Conditioned media was concentrated by centrifugation (Millipore, Billerica, MA, USA) and a protein assay was performed (BioRad, Hercules, CA, USA). 1 mg of protein was pre-cleared with protein A beads after which 1 μg of hsp90α antibody (Assay Designs, Farmingdale, NY, USA) was added to the samples. Samples were washed with RIPA B buffer (50 mM Tris, 150 mM NaCl, 0.5% NP40, 0.25% DOC) boiled, subjected to SDS PAGE, stained with Coomassie Blue and removed from the gel for mass spectrometry analysis. The excised gel bands were analyzed by mass spectrometry as previously described [37]. Coimmunoprecipitation was performed as described above using 2.5 μg of LOXL2 Ab (R&D Systems, Minneapolis, MN, USA) was added to the samples. Samples were washed with RIPA B buffer, boiled and subjected to SDS-PAGE for western blotting. Blots were probed with mouse anti-HSP90alpha (Enzo Life Sciences, Farmingdale, NY, USA) and rabbit anti-LGALS3BP (Thermo Scientific, Tewkbury, MA, USA) primary and goat anti-rabbit and anti-mouse HRP-conjugated (GeneTex, Irvine, CA, USA and Enzo Life Sciences) secondary antibodies for blotting.

### 3.3. Hsp90 Binding and HER2 Degradation Assays

STA-12-7191 binding to Hsp90 alpha was determined using an Hsp90α assay kit from BPS Bioscience San Diego, CA, USA, Cat #50294) containing Hsp90alpha recombinant enzyme, FITC-labeled geldanamycin, assay buffer and a low binding 384-well plate. Binding was assessed using fluorescence polarization using a PHERAstar fluorescence plate reader (BMG Labtech, Ortenberg, Germany). HER2 degradation was assessed in BT474 breast cancer cells (ATCC# HTB-20) using Anti-HER2/neu FITC (BD Biosciences, San Jose, CA, USA) and assessing fluorescence as in the Hsp90 binding assay.

### 3.4. Drug Toxicity and Viability Assays

For each cell line tested, 7 × 10^3^ cells were plated into the central 60 wells of a 96-well tissue culture plate, and allowed to adhere for 24 h. Culture media was then replaced with media containing the drug, either ganetespib or STA-12-7191, with each concentration repeated in triplicate wells. Cells were maintained in drug-containing media for five days, with a media change on day three. Cell viability was then determined using the CellTiter-Glo assay kit (Promega, Madison, WI, USA). Luminescence values were normalized to vehicle control-treated cells. LD_50_ values were calculated and dose-response curves generated using the R statistical software package (R Core Team, 2013) using the *drc* library [38].

### 3.5. Wound Healing Assays

1.5 × 10^5^ MDA-MB231 breast cancer cells or 0.7 × 10^5^ A172 glioma cells were plated in an eight well chamber slide. Cells were allowed to adhere for 24 h and then wounded by scratching a sterile yellow pipette tip length-wise along the chamber. The cells were washed once with 1× PBS and growth media was placed in each well with DMSO (vehicle control), 10 nM ganetespib, or 10 nM STA-12-7191, or 100 nM STA-12-7191. Images were taken immediately after wounding (0 h) and 16 h after wounding. Wound width was calculated using Spot software.

### 3.6. Membrane Permeability Assays

Permeability for STA-12-7191 was conducted using Parallel Artificial Membrane Permeability Assay (BD Biosciences). In this assay the compounds were dissolved n DMSO, diluted with PBS and added to donor wells. The acceptor wells contained PBS only. Samples were incubated for 5 h at room temperature and samples were taken from both the acceptor and donor wells for analysis by LC/MS-MS. Caffeine was used as the positive control and furosemide was used as the negative control.

## 4. Conclusions

These studies introduce two new binders of eHsp90 in MDA-MB231 conditioned media: LOXL2 and G3BP and are consistent with LOXL2 but not G3BP as a potential client of eHsp90. They also test for the first time STA-12-7191 an impermeant inhibitor of Hsp90 derived from ganetespib and show this compound is not only markedly less cytotoxic than ganetespib but it can also inhibit cancer cell migration in a wound healing assay.

While the mass spectrometry analysis suggests that LOXL2 and G3BP associate with eHsp90 in conditioned media by mass spectrometry however, we only have evidence by immunoblot analysis for LOXL2 binding to eHsp90. Furthermore, addition of LOXL2 protein to the wound healing assay increases wound closure thereby compensating for STA-12-7191 inhibition of wound closure. Thus our findings support a role for LOXL2 (but not G3BP) as a potential client for eHsp90 and its established role in cancer makes it a potentially important one. To establish that LOXL2 is a *bona fide* eHsp90 client, it remains to be determined the mechanism by which these proteins are activated and their relevant substrates for invasion. While the mechanism of how LOXL2 acts in eHsp90 stimulated cancer cell migration is not clear but it has many functions that could promote cell migration. The lysyl oxidase activity of LOXL2 can crosslink ECM and inhibiting LOXL2 can markedly decrease crosslinked collagen in the stroma [39]. It is a substrate for MMP2 and MMP9 both of which are activated by eHsp90 [2,9]. An additional factor in LOXL2’s increase of cell motility and invasion is its ability to increase Focal Adhesion Kinase (FAK) phosphorylation leading to increases in focal adhesion dynamics [24,40]. LOXL2 also binds and regulate Snail during transcriptional regulation (intracellular roles) affecting EMT [26] but our IP/MS analyses were conducted on extracellular proteins and we will restrict the studies in this application to LOXL2’s extracellular roles. Future studies will be necessary to determine the mechanism of how LOXL2 is enhancing cellular migration as well as to determine if the mechanism is solely dependent on the extracellular activity of Hsp90.

Hsp90 is an established cancer target with over twenty ongoing clinical trials testing the benefit of Hsp90 inhibitors in cancer treatment. For example, early results are promising from Synta Pharmaceutical’s phase 2 study of ganetespib in combination with docetaxel for advanced stage lung adenocarcinoma and this has led to a current phase-3 clinical trial [17]. Hsp90 has many intracellular functions and there may be toxicities associated with inhibiting all Hsp90 functions as suggested by the 10-fold lower LD_50_ for ganetespib compared to STA-12-7191. While well tolerated, dosing is limited due to intracellular Hsp90 inhibition of normal cells. If a non-cytotoxic extracellular small molecule inhibitor could be employed, this might serve as a compound to inhibit cancer cell migration to reduce cancer spread *in vivo*. This study investigates STA-12-7191 as an alternative candidate impermeant Hsp90 inhibitor that may have advantages over DMAG N-oxide and 4c5, which have been explored in metastasis models in mice. STA-12-7191 has advantages as a small molecule and is likely to be better tolerated *in vivo* than ganetespib. As STA-12-7191 does not readily penetrate the cell membrane, it is likely to be even less cytotoxic although this needs to be demonstrated in a pharmacotoxicity study. Such an impermeant eHsp90 inhibitor would avoid cytotoxicities associated with total Hsp90 inhibition and may permit higher dosing with diminished side effects [6].

While previous studies suggest that inhibiting eHsp90 has benefit in reducing metastasis in two mouse models for melanoma [31] and breast cancer [14] their use for clinical applications may be limited. DMAG N-oxide (derived from 17AAG) is degraded in serum to DMAG, which has retinal toxicity issues [32]. MC5 is an anti-Hsp90 monoclonal antibody has promise but is expensive to prepare in amounts required for clinical trials and has not yet been examined for toxicity. Secondly, these studies used tail vein injection of human cancer cell lines, which really only assesses the later stages of metastasis (survival in circulation, implantation and homing) and also have an additional complication of using human cells homing to mouse organs. Thus far, the *in vitro* data from this study and others on eHsp90 implicate its role is in migration and invasion, the first steps of metastasis. How eHsp90 acts on homing and other later events in metastasis is not clear therefore *in vivo* models that directly test eHsp90’s role in invasion are required.

The data presented herein introduce STA-12-7191 as an alternative to ganetespib. Future studies will be valuable in understanding the mechanism by which STA-12-7191 inhibits cellular migration, and if in fact, that mechanism differs from ganetespib migration inhibition. It will be imperative to examine STA-12-7191 in metastasis models that interrogate invasion as an early step in metastasis. While this study was focused on cells derived from breast cancer and glioblastoma, eHsp90 has also been found for other cancer cells including prostate and melanoma so it is possible that impermeant Hsp90 inhibitors may also have application to reduce invasion for these cancers.
